# A Contemporary Design Process for Single-Phase Voltage Source Inverter Control Systems

**DOI:** 10.3390/s22197211

**Published:** 2022-09-23

**Authors:** Krzysztof Bernacki, Zbigniew Rymarski

**Affiliations:** Department of Electronics, Electrical Engineering and Microelectronics, Faculty of Automatic Control, Electronics and Computer Science, Silesian University of Technology, Akademicka 16, 44-100 Gliwice, Poland

**Keywords:** voltage source inverter, coefficient diagram method, passivity based control, SISO control, MISO control, real-time interface

## Abstract

This paper presents an overview of contemporary voltage source inverter control system design. Design begins with the theoretical considerations that lead to the creation of the system’s differential control law. This stage does not include scaling coefficients for the output voltage, output current, and filter inductor current. Following this, the inverter is modelled in MATLAB’s Simulink environment with an appropriate load and control system. If the resultant simulation provides satisfactory results, a hybrid system consisting of MATLAB’s Simulink and dSpace libraries with the MicroLabBox device is used to interface the simulation with an experimental hardware model in real-time. This allows the hardware plant and measuring traces to be validated. ControlDesk is used to scale the relevant coefficients. During the final stage of the design process, a microprocessor is programmed to control the inverter according to the dSpace simulation results. This requires new scaling values. Throughout every stage of the design process, too high a value of the modulation index disables the reduction of output voltage distortions. This paper details the entire design process for both single-input and multi-input control systems, explaining the scaling process and the required software. Such a modern design process ensures the shortest time between conceptualization and the final product.

## 1. Introduction

The design of a voltage source inverter (VSI) control system begins with a theoretical description of the differential control law that governs the system. The control system should then be verified via simulation (the standard approach is to use MATLAB’s Simulink environment) before finally being implemented on the microprocessor or FPGA system of the experimental VSI. Continuous control laws require further discretization so better is to use the discrete control laws at the beginning. Following validation of the experimental VSI, the final product can be realized. Ideally, this approach is fast and effective. However, following theoretical calculations the output voltage, output current, and filter inductor current scaling factors remain undetermined. These factors all affect the coefficients within the control law. The scaling of voltages and currents in simulation is straightforward. The reference voltage amplitude is defined as unity, and all voltage and current measurements are divided by the input DC voltage. Contemporary design methodologies feature one additional step. Via a MicroLabBox-RTI1202 real-time interface hardware, the dSpace software (including libraries) can be used to drive the experimental VSI using the Simulink model. Throughout all stages of the design process, too high a value of the modulation index disables output voltage distortions from being reduced. The pulse-width modulation (PWM) modulator can become saturated during dynamic increases of the load [1]. However, a modulation index that is too low decreases the efficiency of the VSI. To strike a balance, the modulation index is set to 60% throughout this paper. The literature contains many examples of the use of dSpace with different real-time interfaces in power electronic systems [2,3,4]. However, this paper demonstrates the entire process by which dSpace is used with a real-time hardware interface during the design process of a VSI. When using MicroLabBox the scaling process is similar to that of the final microprocessor controller, because the voltage and currents are amplified within the experimental VSI device and the hardware VSI plant is controlled. Furthermore, the reference sinusoidal waveform amplitude that corresponds to a modulation index of 100% depends on the PWM modulation scheme [5,6,7]. The reference waveform amplitude takes the value of unity for the Simulink modulator, and 0.5 for the dSpace modulator used for the first modulation scheme in this paper. When subject to microprocessor control, the amplitude depends on the quotient of the PWM unit comparator input frequency and the switching frequency (in the presented experiment it is 1640). The final step of the design process is the implementation of the controller on the microprocessor. Other than when using Simulink, this requires further scaling of the current versus voltage measurements. The scaling process requires dedicated software: dSpace requires ControlDesk; microprocessor control requires dedicated PC software that can support data exchange with the inverter via a USB port. An additional problem is the evaluation of a Bode plot of the measurement traces [8,9]. This is typically modelled within a frequency range lower than the resonant frequency of the output filter as a simple delay, with one switching period for the amplifiers and one switching period for the PWM modulator [10].

**Figure 1:**
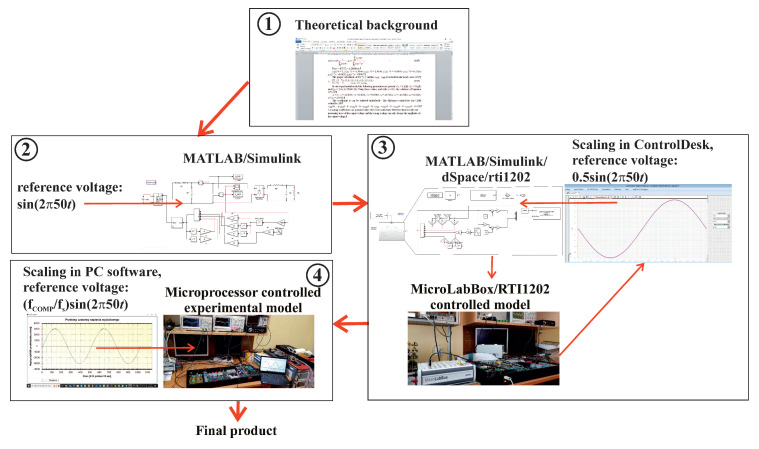
Flowchart of the modern VSI controller design process.

The objective of this paper is to provide a detailed account of the contemporary design of VSI control systems. This process will be demonstrated using two examples. The first example is a simple single input single output (SISO) control system that uses the discretized coefficient diagram method (CDM) [10,11,12,13,14,15,16], requiring only a single input variable: output voltage. The second example is a more complex multiple input single output (MISO) control system using passivity-based control (PBC) [10,11,12,17,18,19,20], with measurements of the output voltage, output current, and inductor current. Specifically, this paper uses improved PBC v2 (IPBC2) [10]. Figure 1 presents the entire VSI controller design process, from the theoretical description to the final product. This includes the use of MATLAB’s Simulink environment, the combination of dSpace and ControlDesk via a MicroLabBox-RTI1202 real-time interface, Keil μVision C++, dedicated PC software written in C#, and the experimental model with a STM32F407VG microprocessor. 

The novelty of the paper is:

1. Presentation of the full process of design–from a theoretical background, through the simulation, using real time interface (RTI) and dSpace libraries up to the final stage of the design process–programming the microprocessor that will control the VSI. Using real time interface creates design much more flexible.
2. Definition of requirements of the design process final success:
  (a) The control law should be realized in a similar way in all the stages of the design process. It means that in the simulation the input controller data and supply of the reference waveform was measured using a sample time equal to the switching period. The PWM modulator should have the sample time equal to the period of the waveform on the input of the microprocessor PWM unit comparator (the much higher frequency than the switching frequency).
  (b) The real time interface and the microprocessor should use the same software architecture based on interrupts (trigger events in case of the RTI) from the PWM modulator.
  (c) The scaling procedure is crucial because wrong scaling changes the control law coefficients. In none of the referred papers [2,3,4] concerning the RTI usage, the scaling procedure of voltages and currents is presented. In [3] where the RTI–MicroLabBox and dSpace software was used there is nothing about using ControlDesk–the software that enables scaling.

Scaling the microprocessor controller requires data transfer from the PC and using specialized software to visualize the measured values scaled in units of the analog-to-digital converter used in the microprocessor. The scaling procedure depends on the ratio of the PWM modulator comparator input frequency and the switching frequency. It is described in detail in the presented paper. 

This paper will be useful for engineers and researchers who design VSIs, by presenting them the novel suite of design tools and techniques that are required, in addition to instructions on their application. Using RTI (MicroLabBox with dSpace) makes the design process more flexible and faster.

Section 2, Section 3, Section 4 and Section 5 present the design process for SISO CDM control, and Section 6, Section 7, Section 8 and Section 9 present the design process for MISO PBC control. The control results are presented and compared using the total harmonic distortion (THD) of the VSI output voltage for a nonlinear rectifier RC load with power factor *PF* = 0.7. 

## 2. Theoretical Background of SISO CDM Control Materials

**Figure 2:**
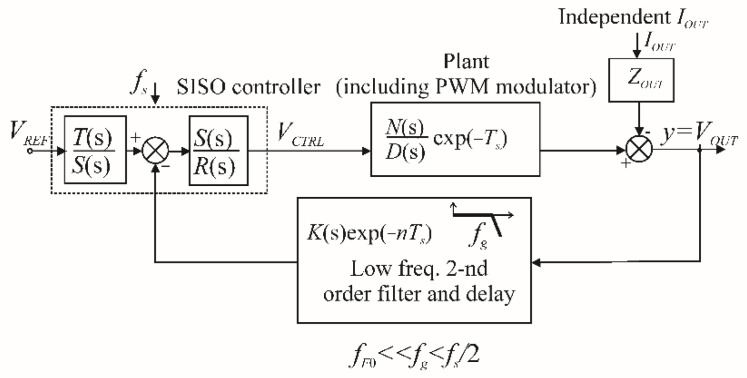
SISO control of a VSI.

Figure 2 shows a VSI with a SISO controller. The output current is treated as an independent disturbance or the state variable, with the same result in both cases [11,16,20,21,22,23,24]. 

One of the simplest control designs is Manabe’s CDM controller [13,14,16], which uses *T*, *S*, and *R* polynomials. In its most basic form, the coefficients of the closed-loop characteristic equation are calculated from Manabe standard form. These coefficients provide the control for the time constant *τ* of the closed-loop system. The output voltage of a closed loop system with the output current treated as an independent disturbance is given by (1):

(1) $$v_{OUT}(s)=\frac{T(s)N(s)}{R(s)D(s)+S(s)N(s)}v_{REF}(s)-\frac{Z_{OUT}R(s)D(s)}{R(s)D(s)+S(s)N(s)}I_{OUT}(s)$$

where *N(s)* contains all the loop delays. The characteristic equation of a closed-loop system is given by (2):

(2) $$P(z^{-1})=R(z^{-1})D(z^{-1})+S(z^{-1})N(z^{-1})=\sum_{i=0}^{n}p_{zi}z^{-i}.$$

To calculate the controller parameters, a model of the inverter plant is required [6]. For this paper, the inverter plant was modelled as an output *L_F_C_F_* filter described by the assigned state variables (3):

(3) $$x=[\begin{array}{ccc} v_{OUT} & i_{LF} & i_{OUT}{]}^{T} \end{array},$$

and the state Equation (4):

(4) $$\dot{x}=Ax+Bu,$$

where matrix **A** and **B** are given by (5):

(5) $$A=\left[\begin{array}{ccc} 0 & \frac{1}{C_{F}} & -\frac{1}{C_{F}}\\ -\frac{1}{L_{F}} & -\frac{R_{LF}}{L_{F}} & 0\\ 0 & 0 & 0 \end{array}\right]\;,\;B=\left[\begin{array}{c} 0\\ \frac{1}{L_{F}}\\ 0 \end{array}\right].$$

**Figure 3:**
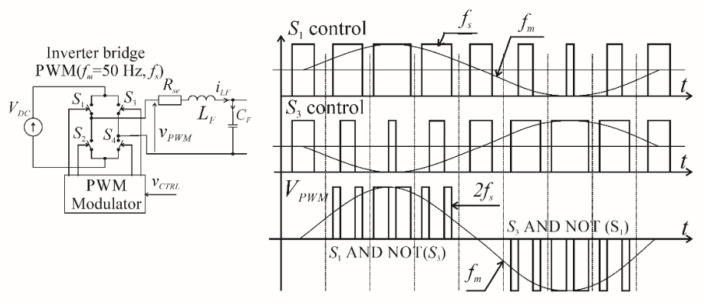
Overview of the first PWM modulation scheme.

The state Equation (4) are solved during a single *k*-th switching period *T_s_*, for double edge three-level PWM, with a switching-on time period *T_ONk_*. The solution of the state space equations depends on the type of modulation—double edge, three-level modulation was chosen as the most suitable for a four-transistor bridge. Some schemes of this type of modulation are presented in [5,6,7]. This paper uses the first presented scheme, as this is most appropriate for instantaneous control. An overview of the scheme is shown in Figure 3. The advantages of this controller include the possibility of controlling output voltage when crossing zero, and an output switching frequency double that of the transistor switching frequency.

Each transistor within the two legs of the H-bridge is switched with frequency *f_s_*. However, the final switching frequency of the output waveform is 2*f_s_*, due to the current flowing through the pairs of switches connected in series: *S*_1_ and *S*_4_ or *S*_3_ and *S*_2_. This results in two output pulses during a single switching period. Within the modulation scheme the control of the switches can be described analytically as (6)–(9):

(6) $$S_{1}:T_{ON}(k)/T_{s}=0.5M\sin(k\frac{2\pi }{f_{s}/f_{m}})+0.5M,$$

(7) $$S_{2}:NOT(S_{1}),$$

(8) $$S_{3}:T_{ON}(k)/T_{s}=0.5M\sin((k\frac{2\pi }{f_{s}/f_{m}})+\pi )+0.5M,$$

(9) $$S_{4}:NOT(S_{3}),$$

where *T_ON_* is the switching on time during a single switching period *T_s_ = 1/f_s_*, and *k* = 0 … (*f_s_*/*f_m_* −1) and *f_s_*/*f_m_* is an integer.

Solving the state space equations provides the exponential function **x***_k+_*_1_ of *T_ONk_*, which can then be linearized [6]. This gives the discrete linear state Equation (10):

(10) $$x_{k+1}=A_{D}x_{k}+G_{D}T_{ONk},$$

where the state matrix **A***_D_* and the state matrix **G***_D_* are given by (11), (12):

(11) $$A_{D}=e^{AT_{s}}=\Phi (T_{s})={L^{-1}[{\left(sI-A\right)}^{-1}]|}_{t=T_{s}},\;A_{D}=\Phi (T_{s})=\left[\begin{array}{ccc} \phi_{11} & \phi_{12} & \phi_{13}\\ \phi_{21} & \phi_{22} & \phi_{23}\\ \phi_{31} & \phi_{32} & \phi_{33} \end{array}\right],$$

(12) $$G_{D}=e^{AT_{s}/2}BV_{DC}=\Phi (T_{s}/2)BV_{DC},\;G_{D}=\left[\begin{array}{c} g_{11}\\ g_{21}\\ g_{31} \end{array}\right],$$

with coefficients *ϕ_ij_* (13) and *g_i_*_1_ (14):

(13) $$\xi_{F}=\frac{1}{2}R_{se}\sqrt{\frac{C_{F}}{L_{F}}},\;\omega_{F0}=\frac{1}{\sqrt{L_{F}L_{F}}},\;\phi_{11}=[\cos\left(\omega_{F0}T_{s}\right)+\xi_{F}\sin\left(\omega_{F0}T_{s}\right)]\exp(-\xi_{F}\omega_{F0}T_{s}),\;\phi_{12}=\frac{1}{\omega_{F0}C_{F}}\sin(\omega_{F0}T_{s})\exp(-\xi_{F}\omega_{F0}T_{s}),\;\varphi_{13}=-\varphi_{12}+R_{LF}(\varphi_{11}-1),\;\phi_{21}=-\frac{C_{F}}{L_{F}}\varphi_{12},\;\phi_{22}=[\cos(\omega_{F0}T_{s})-\xi_{F}\sin(\omega_{F0}T_{s})]\exp(-\xi_{F}\omega_{F0}T_{s}),\;\phi_{23}=1-\varphi_{11},\;\phi_{31}=0,\;\phi_{32}=0,\;\;\phi_{33}=1.$$

(14) $$g_{11}=V_{DC}\omega_{F0}\sin(\omega_{F0}\frac{T_{s}}{2})\exp(-\xi_{F}\omega_{F0}\frac{T_{s}}{2}),\;g_{21}=\frac{V_{DC}}{L_{F}}[\cos(\omega_{F0}\frac{T_{s}}{2})-\xi_{F}\sin(\omega_{F0}\frac{T_{s}}{2})]\exp(-\xi_{F}\omega_{F0}\frac{T_{s}}{2}),\;g_{31}=0.$$

For a double edge PWM and a digital modulator implementing all the required loop delays, the VSI gain is given by (15):

(15) $$K_{VSI}=\frac{N(z^{-1})}{D(z^{-1})}=\frac{a_{2}z^{-2}+a_{3}z^{-3}}{1+b_{1}z^{-1}+b_{2}z^{-2}},$$

where (16):

(16) $$a_{2}=\frac{T_{s}}{V_{DC}}g_{11},\;a_{3}=\frac{T_{s}}{V_{DC}}(\varphi_{12}g_{21}-\varphi_{22}g_{11})\;,\;b_{1}=-(\varphi_{11}+\varphi_{22}),\;b_{2}=\varphi_{11}\varphi_{22}-\varphi_{12}\varphi_{21}.$$

For a system that is subject to a disturbance, the degrees of *R* and *S* are greater than or equal to *n* − 1, where *n* is the degree of *D*. The second degree of *S* and the second degree of *R* are given by (17):

(17) $$S(z^{-1})=\sum_{i=0}^{2}s_{i}z^{-i}\;,\;R(z^{-1})=\sum_{i=0}^{2}r_{i}z^{-i},\;r_{0}=1.$$

The underlying objective of CDM control is to obtain the *s_i_* and *r_i_* coefficients are thereby solve the Diophantine Equation (18):

(18) $$\begin{array}{l} (1+r_{1}z^{-1}+r_{2}z^{-2})(1+b_{1}z^{-1}+b_{2}z^{-2})+(s_{0}+s_{1}z^{-1}+s_{2}z^{-2})(a_{2}z^{-2}+a_{3}z^{-3})=\\ 1+\sum_{i=1}^{5}p_{zi}z^{-i} \end{array},$$

which can be written as (19):

(19) $$\left[\begin{array}{ccccc} 1 & 0 & 0 & 0 & 0\\ b_{1} & 1 & a_{2} & 0 & 0\\ b_{2} & b_{1} & a_{3} & a_{2} & 0\\ 0 & b_{2} & 0 & a_{3} & a_{2}\\ 0 & 0 & 0 & 0 & a_{3} \end{array}\right]\left[\begin{array}{c} r_{1}\\ r_{2}\\ s_{0}\\ s_{1}\\ s_{2} \end{array}\right]=\left[\begin{array}{c} p_{z1}-b_{1}\\ p_{z2}-b_{2}\\ p_{z3}\\ p_{z4}\\ p_{z5} \end{array}\right],$$

where the *p_zi_* coefficients are assigned from the Manabe standard form, and it was assumed that *r*_0_ = *p*_0_ = 1. The coefficients *p_i_* of the fifth degree of Manabe standard form for a continuous system are given by

*p*_0_(*s*^0^) = 1, *p*_1_(*s*^1^) = *p*_0_*τ*, *p*_2_(*s*^2^) = 0.4*p*_0_*τ*^2^, *p*_3_(*s*^3^) = 0.08*p*_0_*τ*^3^, *p*_4_(*s*^4^) = 0.008*p*_0_*τ*^4^, *p*_5_(*s*^5^) = 0.0004*p*_0_*τ*^5^,

where *τ* is the time constant of a closed-loop system. For *f_s_* = 25,600 Hz, satisfactory experimental results were obtained with *τ* = 5.5*T_s_*. Lower values of *τ* lead to output voltage oscillations; higher values of *τ* lead to poorer control.

Via the zero-order hold method and a discretization cycle of *T_s_* = 1/25,600 s, the MATLAB *c*2*d* function was used to obtain a discrete-time transfer function (20):

(20) $$K(z)=c2d(\frac{1}{\sum_{i=0}^{5}p_{i}(s)s^{i}},T_{s})=\frac{\sum_{i=0}^{5}w_{i}(z)z^{-i}}{\sum_{i=0}^{5}p_{zi}(z^{-1})z^{-i}}.$$

For *τ* = 5.5*T_s_* (*T_s_* = 1/25,600 s),

*p_z0_*(*z*^0^) = 1, *p*_z1_(*z*^−1^) = −1.9655, *p*_z2_(*z*^−2^) = 1.5925, *p*_z3_(*z*^−3^) = −0.7017, *p*_z4_(*z*^−4^) = 0.1886,

*p*_z5_(*z*^−5^) = −0.0263.

The accurate calculation of *T*(*z*^−1^) = *t*_0_ enables *v_OUT_* = *v_REF_* to be maintained in the steady state (21):

(21) $$t_{0}=\frac{P(z=1)}{N(z=1)}=\frac{V_{DC}}{T_{s}}\frac{1+p_{z1}+p_{z2}+p_{z3}+p_{z4}+p_{z5}}{\varphi_{12}g_{21}+(1-\varphi_{22})g_{11}}.$$

The experimental model used the following parameters: *L_F_* = 2 mH, *C_F_* = 51 μF, *R_se_* = 1 Ω, and *f_s_* = 25,600 Hz. Using these values, and with *τ* = 5.5*T_s_*, the solutions of Equation (19) are

*r*_0_ = 1, *r*_1_ = −0.0299, *r*_2_ = 0.3758, *s*_0_ = 28.0795, *s*_1_ = −20.2981, *s*_2_ = −3.6181, *t*_0_/*V_DC_* = 5.5090.

The coefficient *t*_0_ can be adjusted individually and is multiplied by the modulation index *M*. This should always be less than unity to allow for the rapid increase of the voltage in the input of the output filter. The difference control law for CDM control is given by (22):

(22) $$\begin{array}{ll} v_{CTRL}(k)= & -r_{1}v_{CTRL}(k-1)-r_{2}v_{CTRL}(k-2)+t_{0}v_{REF}-\\ & s_{0}v_{OUT}(k-1)-s_{1}v_{OUT}(k-2)-s_{2}v_{OUT}(k-3) \end{array},$$

which contains no scaling coefficient. The scaling coefficients will be further incorporated into Equation (22).

## 3. MATLAB’s Simulink Simulation of SISO CDM Control

**Figure 4:**
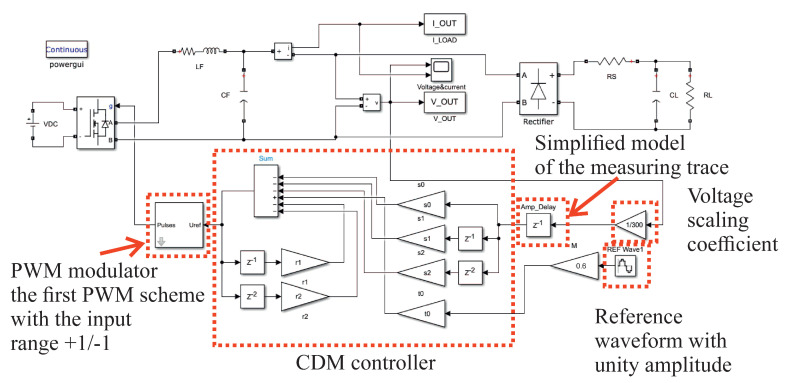
The MATLAB’s Simulink model of the VSI with SISO-CDM control.

**Figure 5:**
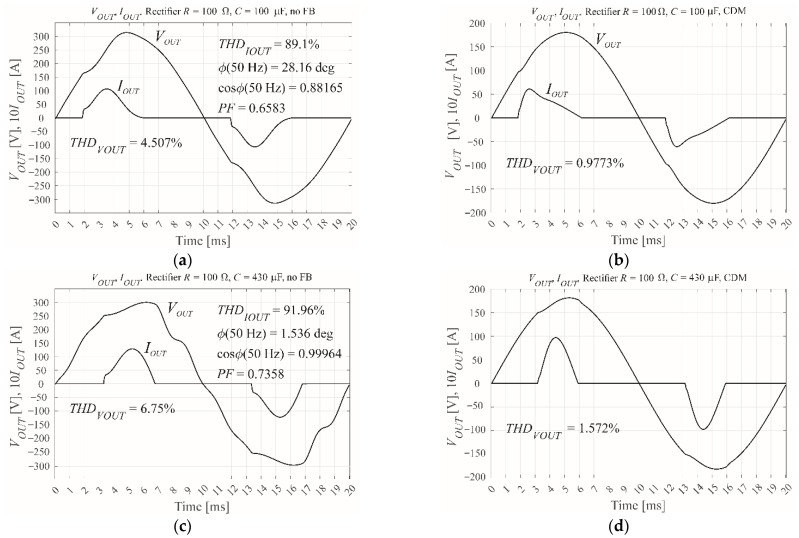
The simulated output voltage and current for the VSI when subject to a nonlinear rectifier RC load, showing (**a**) open loop with *R* = 100 Ω, *C* = 100 μF, and *PF* = 0.7, (**b**) CDM control with *R* = 100 Ω, *C* = 100 μF, and *M* = 0.6, (**c**) open loop with *R* = 100 Ω, *C* = 430 μF, and *PF* = 0.7, and (**d**) CDM control with *R* = 100 Ω, *C* = 430 μF, and *M* = 0.6.

As shown in Figure 4, the controller was modelled in the Simulink environment of MATLAB R2021b. The Simulink model was tested with the calculated scaling coefficients. The simulation results are shown in Figure 5. The scaling coefficient is simply 1/*V_DC_*, because the reference sinusoidal waveform sin(2π50*t*) has a unity amplitude. The PWM modulator unit has an input range of ±1. The output voltage measuring trace is modelled as a single switching period delay [10], with the PWM modulator contributing an additional delay of *T_s_*. The most demanding test load is the nonlinear rectifier RC load (Figure 5c,d present it for *R* = 100 Ω, *C* = 430 μF, when *PF* = 0.7). This is defined by the EN 62040 standard [25] as the most common load for an uninterruptible power supply with an output power of less than 3 kW. Figure 5a,b show a less demanding nonlinear load with *R* = 100 Ω and *C* = 100 μF.

The performance of the control is estimated by the THD of the output voltage (Figure 5), with the operation of the VSI under CDM control (Figure 5b,d) being compared with its open loop operation (Figure 5a,c). The CDM controller was tested using a relatively low modulation index of *M* = 0.6 to prevent the saturation of the modulator that can occur for higher values of *M*. The same value will be used throughout this paper.

## 4. Interfacing MATLAB’s Simulink and dSpace Simulation of the SISO CDM Controller with the Experimental Model

Following initial simulations in Simulink, the MicroLabBox RTI1202 real-time interface was used to interface dSpace simulation blocks with the experimental model. To this end, the dSpace RTI1202 FPGA and dSpace RTI Electric Motor Control Blockset libraries were used. The compiled simulation was automatically loaded onto the MicroLabBox FPGA to provide high speed data conversion and computation with little time delay. The simulation should be designed to imitate the microprocessor procedure as closely as possible. The microprocessor control software used an infinite main loop (defined using while (1)), with the “watchdog” and all functions are handled by PWM interrupts which fetch the analogue-to-digital converter (ADC) values of the output voltage. In the dSpace simulation, the interrupts are represented by Trigger line 1 events from an EMC Multichannel PWM block, which are handled by an ADC Class 1 Hardware Interrupt block. This Hardware Interrupt (HWINT) block is connected to the input port of the Function-Call Subsystem, which contains all the components of the inverter control blocks, including the EMC Multichannel PWM block and the ADC Class 1 block. The sample time of each of these blocks is inherited from the PWM block triggering event. In a similar manner to the microprocessor software, the switching frequency is the input of the PWM block. 

**Figure 6:**
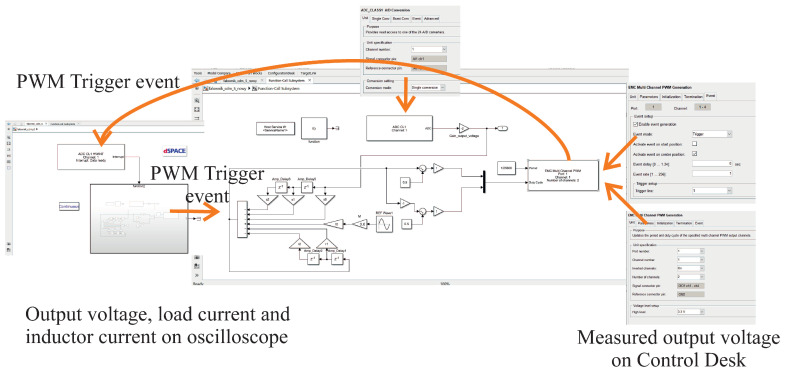

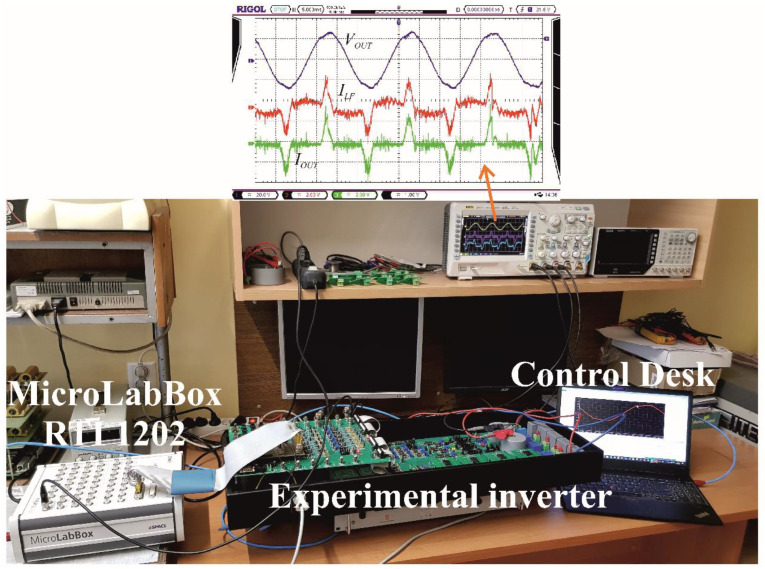
The real-time interface of the dSpace simulation blocks and the experimental inverter using MicroLabBox RTI1202.

Once loaded with the control software, MicroLabBox can drive the experimental inverter using four DIO Class 1 3.3 V digital outputs, operating on channels 1–4. MicroLabBox receives the measured output voltage via the ADC Class 1 channel 1, with a single conversion (−10–+10 V input range) following the trigger event from the PWM block. The PWM block is configured to drive a block of four transistors with inverting signals for the low transistors. A 500 ns dead time is implemented for the experimental inverter. For the case of inverted channels set as active, the block automatically reserves the same number of channels for inverted signals as specified for non-inverted signals. The first inverted channel is channel 3, corresponding to *S*_2_ in Figure 3. The second inverted channel is channel 4, corresponding to *S*_4_ in Figure 3. The PWM block inputs take values in the range 0–1. Hence, the input waveforms are sinusoidal with an amplitude of 0.5, shifted mutually 180 degrees in phase and both raised 0.5 with zero level. The generation of the two shifted strings for the PWM block inputs is presented in Figure 6. The measured output voltage waveforms are visualized via ControlDesk, which is part of the dSpace software package. The output voltage is scaled by using the Time Plotter feature of ControlDesk to compare two waveforms. For an open loop system and a nominal resistance load of 50 Ω, the measured output voltage should be given by the reference waveform 0.5sin(2π50*t*). Once these waveforms have been equalized, by changing the gain of the output voltage, the output voltage gain value is set as a scaling coefficient. The measuring trace can reverse the sign of the signal (in the experimental model), so the sign must be set correctly. With a modulation index of *M* = 0.6, it was found an output voltage scaling coefficient of −2. 

**Figure 7:**
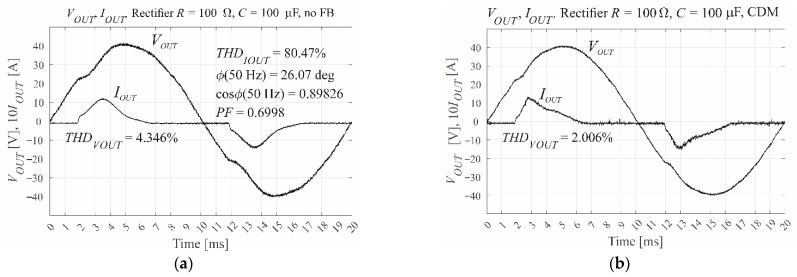

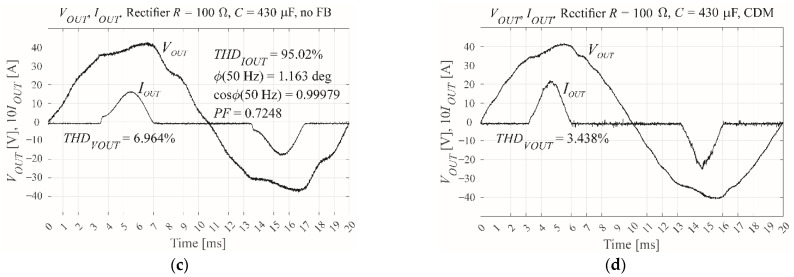
The measured output voltage and current of the experimental VSI using MicroLabBox real time interface when subject to a nonlinear rectifier RC load, showing (**a**) open loop with *R* = 100 Ω, *C* = 100 μF, and *PF* = 0.7, (**b**) CDM control with *R* = 100 Ω, *C* = 100 μF, and *M* = 0.6, (**c**) open loop with *R* = 100 Ω, *C* = 430 μF, and *PF* = 0.7, and (**d**) CDM control with *R* = 100 Ω, *C* = 430 μF, and *M* = 0.6.

Figure 7 shows the results of CDM control via the MicroLabBox, versus open loop control of the same system. Both approaches use a nonlinear rectifier *RC* load with100 μF or 430 μF, 100 Ω, and *PF* ≈ 0.7. The results show a change in the shape of the load current, with the current loading forced to the load capacitor. A lower filter inductor value *L_F_* would produce a smaller THD coefficient.

## 5. Implementation of the CDM Controller in the VSI Microprocessor

**Figure 8:**
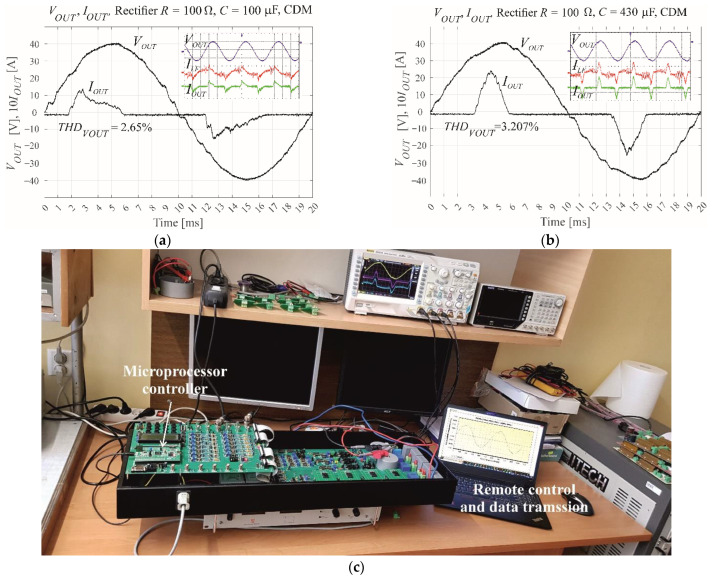
Waveforms of the output voltage, output current, inductor current and measurement of the output voltage THD for CDM and (**a**) a nonlinear rectifier RC load with *R* =100 Ω, *C* = 100 μF, and *M* = 0.6, and (**b**) a nonlinear rectifier RC load with *R* =100 Ω, *C* = 430 μF, and *M* = 0.6, in addition to (**c**) an image of the experimental environment showing the VSI and the microprocessor controller.

The final step of the design process was to implement the validated controller on the STM32F407VG microprocessor. The microprocessor code was written in Keil μVision 5 C++. As described in Section 4, the main function of the code consists of an infinite loop, with functions called by an event handler that waits for PWM unit interrupts. Hence, the control process is identical to that provided by MicroLabBox. However, the two approaches differ in terms of the scaling of the output voltage measuring trace. The dedicated PC application that handles data transmission, data visualization, and communication with the microprocessor-controlled inverter via USB port was written in C#. The purpose of this application is analogous to the role played by the ControlDesk software for the MicroLabBox controlled system. However, in the older solutions, it was possible to use the digital-to-analogue converter implemented in the microprocessor to visualize on the oscilloscope the internal waveforms from the microprocessor without the dedicated PC software. The reference voltage takes the form 0.5*f_COMPmax_*/*f_s_*sin(2π50*t*), where *f_COMPmax_* is the maximum frequency on the input of the PWM unit comparator and *f_s_* is the switching frequency. Hence, the peak-to-peak amplitude of the reference voltage is given by *f_COMPmax_*/*f_s_*. For the experimental inverter, *f_COMPmax_*/*f_s_* = 84 MHz/25,600 Hz ≈ 3281. Therefore, the maximum amplitude of the reference waveform was 1640. The 13-bit (12 bits plus the sign) ADC controller allowed measurement in the range −4095–4095. Using the visualization provided by the PC application, the hardware gain of the voltage measurement trace was adjusted to a nominal output voltage amplitude of 3000 units—Greater than the reference amplitude of 1640 units. This provided more accurate measurement across the entire ADC range. Finally, the voltage gain scaling coefficient *g_v_* should be 1640/3000. Again, a modulation index of *M* = 0.6 was used. Figure 8 shows the output current and voltage and inductor current waveforms when using microprocessor control, in addition to an image of the experimental setup.

## 6. Theoretical Background of MISO PCB Control

Without direct measurement of the output current (an independent disturbance), SISO control is unable to precisely control the output voltage in the case of large, rapid changes in the output current for a standard [25] nonlinear rectifier RC load. This functionality is provided by MISO PCB control. For IPBC2, the output voltage, output current, and inductor current are input variables of the controller.

**Figure 9:**
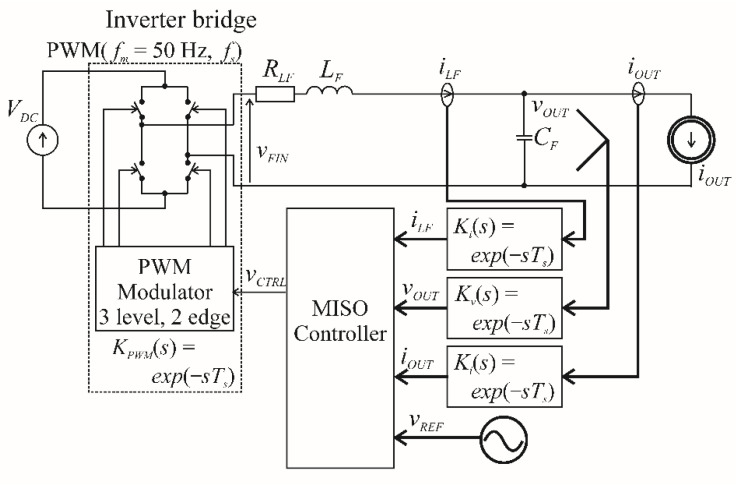
MISO control of a VSI.

Figure 9 models the control of a VSI by MISO. It was shown in [10] that each of the experimental model’s measurement traces can be approximately modelled as a single switching period delay. For the described system, this delay had a value of 39 μs. The additional delay is implemented by the PWM modulator, with the data stored in its registers during the *k*th period controlling the width of the pulses during the *k*+1th period. Two different MISO PBC controllers were tested: one that did not account for the double switching period delay, and one that made a simplified prediction of the state variables in subsequent periods using the discrete model of a VSI [12]. There was no noticeable difference between the two controllers in terms of the quality of the VSI output voltage at a relatively high switching frequency of *f_s_* = 25,600 Hz, and with *C_F_* = 50 μF. The simplified approach is sufficient for the presentation of the VSI control design methodology. The load current *i_OUT_* is treated as the independent disturbance and is modelled as the current source.

The central principle of PBC is that the system is stable if it is passive. The system is passive if the energy supplied to it exceeds the stored energy. Energy is stored within two non-dissipative components—The filter coil and the filter capacitor. The energy stored within a system is described by the Hamiltonian function *H*(*x*) (also known as the Lyapunov function [18]). The Hamiltonian function of the error vector **e** is (23):

(23) $$H(e)=\frac{1}{2}(L_{F}{\left(i_{LF}-i_{LFref}\right)}^{2}+C_{F}{\left(v_{OUT}-v_{OUTref}\right)}^{2})=\frac{1}{2}e^{T}P^{-1}e,$$

where

(24) $$e=\left[\begin{array}{c} L_{F}(i_{LF}-i_{LFref})\\ C_{F}(v_{OUT}-v_{OUTref}) \end{array}\right],\;P^{-1}=\left[\begin{array}{cc} 1/L_{F} & 0\\ 0 & 1/C_{F} \end{array}\right].$$

The equilibrium of a closed-loop system is asymptotically stable [19] if *H*(**e**) has a minimum at **x** = **x**_ref_ (25):

(25) $${\frac{\partial H(e)}{\partial x}|}_{x=x_{ref}}=0,\;{\frac{\partial^{2}H(e)}{\partial x^{2}}|}_{x=x_{ref}}>0,\;\mathrm{where}\;x={\left[\begin{array}{cc} L_{F}i_{LF} & C_{F}v_{OUT} \end{array}\right]}^{T}.$$

The system is passive if the time derivative of *H*(**e**) is negative (26):

(26) $$\frac{dH(e)}{dt}<0.$$

The control law of IPBC2 for single-phase inverters is based on the control law for interconnection and damping assignment PBC (IDAPBC) [17,19,20]. The equation for a closed loop PBC system is given by (27):

(27) $$\dot{e}=[J-(R+R_{a})]P^{-1}e.$$

The equation for an open loop system is given by (28):

(28) $$\dot{x}=[J-R]P^{-1}x+\left[\begin{array}{c} V_{DC}\\ 0 \end{array}\right]m+\left[\begin{array}{c} 0\\ -1 \end{array}\right]i_{OUT}.$$

The IPBC2 control law is given by the difference between the closed loop and open loop Equation (29):

(29) $$\dot{e}-\dot{x}=[J-R]P^{-1}(e-x)-R_{a}P^{-1}e-\left[\begin{array}{c} V_{DC}\\ 0 \end{array}\right]m-\left[\begin{array}{c} 0\\ -1 \end{array}\right]i_{OUT}.$$

The interconnection matrix **J**, the damping matrix **R**, and the PBC controller matrix **R***_a_*, are defined as (30):

(30) $$J=\left[\begin{array}{cc} 0 & -1\\ 1 & 0 \end{array}\right],\;R=\left[\begin{array}{cc} R_{LFe} & 0\\ 0 & 0 \end{array}\right],\;R_{a}=\left[\begin{array}{cc} R_{i} & 0\\ 0 & K_{v} \end{array}\right],$$

where *R_i_* is the current error gain, *K_v_* is the voltage error conductive gain, and *R_LFe_* is the serial equivalent resistance of the inverter.

The final form of the IPBC2 control law is then given by (31) and (32):

(31) $$v_{CTRL}(t)=L_{F}di_{LFref}/dt+(R_{LFe}+R_{i})i_{LFref}+v_{OUTref}-R_{i}i_{LF},$$

(32) $$i_{LFref}=C_{F}dv_{OUTref}/dt-K_{v}(v_{OUT}-v_{OUTref})+i_{OUT}.$$

Now consider a difference control law for a single-phase VSI with a PBC that is easy to implement using microprocessor control (33) and (34):

(33) $$v_{CTRL}(k)=-R_{i}i_{LF}(k)+(R_{i}+R_{LFe})i_{LFref}(k)+L_{F}\frac{i_{LFref}(k)-i_{LFref}(k-1)}{T_{c}}+v_{OUTref}(k),$$

(34) $$i_{LFref}(k)=K_{v}[v_{OUTref}(k)-v_{OUT}(k)]+C_{F}\frac{v_{OUTref}(k)-v_{OUTref}(k-1)}{T_{c}}+i_{OUT}(k).$$

This difference control law (Equations (33) and (34)) is used throughout the development of the MISO PCB controller, including MATLAB’s Simulink simulations, the MicroLabBox interfaced dSpace simulations, and the microprocessor control of the VSI.

The values *R_LFe_* + *R_i_* and *K_v_* should be positive. This allows the closed loop IPBC system [10,11] to have roots *λ*_1,2_ with negative real components (35):

(35) $$\begin{array}{l} \lambda_{1,2}=\\ \frac{\left\{-[(R_{LFe}+R_{i})C_{F}+L_{F}K_{V}]\pm \sqrt{{\left[(R_{LFe}+R_{i})C_{F}+L_{F}K_{v}\right]}^{2}-4L_{F}C_{F}[1+(R_{LFe}+R_{i})K_{v}]}\right\}}{2L_{F}C_{F}} \end{array}$$

The real components of these roots are always negative for positive values *R_LFe_* + *R_i_* and *K_v_*. As such, this condition does not provide any upper bounds for current and voltage gains. The higher the gains, the greater the convergence of the error tracking. However, excessively high IPBC2 gain values can cause oscillations of the VSI output voltage. Such oscillations occur when the control voltage increases more quickly than the width of the PWM pulses can change. This creates a saturation-like effect within the control loop. The higher the switching frequency, the higher the speed of the PWM modulator, and hence the maximum acceptable gains [11]. The fastest change in modulation during a single switching period *T_s_* is *V_DC_* (*V_DC_*/*T_s_*). At all times, the delay of the modulator is omitted. During a single sampling period, the approximation d(*v_OUTref_*)/d*t* ≈ 0 can be made. Therefore, from Equation (34) it was obtained (36), (37) and (39):

(36) $$i_{LFref}(kT_{s})\approx K_{v}[v_{OUTref}(kT_{s})-v_{OUT}(kT_{s})]+i_{OUT}(kT_{s}),$$

(37) $$i_{LFref}(kT_{s})\approx (\frac{1}{R_{LOAD}}-K_{v})v_{OUT}(kT_{s})+v_{OUTref}(kT_{s}),$$

(38) $$\frac{di_{LFREF}(kT_{s})}{dt}\approx (\frac{1}{R_{LOAD}}-K_{v})\frac{dv_{OUT}(kT_{s})}{dt}.$$

Correspondingly, from Equation (33) it obtained (39) and (40):

(39) $$\frac{dv_{CTRL}(kT_{s})}{dt}\approx L_{F}\frac{d^{2}i_{LFref}(kT_{s})}{dt^{2}}+(R_{LFe}+R_{i})\frac{di_{LFref}(kT_{s})}{dt}-R_{i}\frac{di_{LF}(kT_{s})}{dt},$$

(40) $$\begin{array}{l} \frac{dv_{CTRL}(kT_{s})}{dt}\approx L_{F}(\frac{1}{R_{LOAD}}-K_{v})\frac{d^{2}v_{OUT}(kT_{s})}{dt^{2}}+\\ (R_{LFe}+R_{i})(\frac{1}{R_{LOAD}}-K_{v})\frac{dv_{OUT}(kT_{s})}{dt}-R_{i}\frac{di_{LF}(kT_{s})}{dt} \end{array}$$

During a single switching cycle, for *R_LOAD_* >> 1/(2π*f_s_C_F_*), the following approximations (41), (42) can be made:

(41) $${\frac{di_{LF}(kT_{s})}{dt}|}_{\max,\min}\approx \pm \frac{V_{DC}}{L_{F}},\;{\frac{dv_{OUT}(kT_{s})}{dt}|}_{\max}\approx \frac{i_{LF}}{C_{F}},\;{\frac{d^{2}v_{OUT}(kT_{s})}{dt^{2}}|}_{\max}\approx {\frac{d}{dt}(\frac{i_{LF}}{C_{F}})|}_{\max}\approx \pm \frac{V_{DC}}{L_{F}C_{F}}$$

(42) $${\left|\frac{dv_{CTRL}(kT_{s})}{dt}\right|}_{\max}\approx K_{v}[L_{F}+(R_{i}+R_{LFe})T_{s}]\frac{V_{DC}}{L_{F}C_{F}}+R_{i}\frac{V_{DC}}{L_{F}}$$

From Equation (43) it was obtained the upper boundary conditions on the gains *R_i_* and *K_v_* (43):

(43) $$K_{v}[1+(R_{i}+R_{LFe})\frac{T_{s}}{L_{F}}]\frac{1}{C_{F}}+R_{i}\frac{1}{L_{F}}<f_{s}.$$

**Figure 10:**
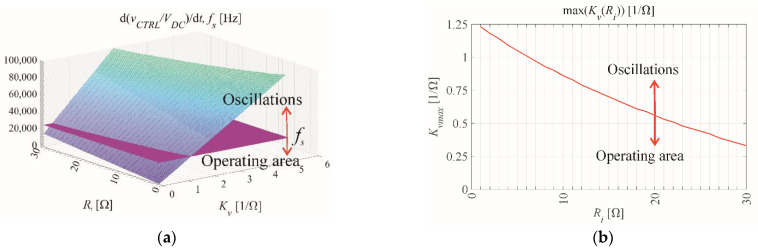
The relationship between maximum voltage gain *K_v_* and current gain *R_i_* for the assigned VSI parameters *f_s_* = 25,600 Hz, *L_F_* = 2 mH, *C_F_* = 51 μF, and *R_LFe_* = 1 Ω in (**a**) 3-D and (**b**) 2-D.

Equation (43) demonstrates the influence of switching frequency *f_s_* = 1/*T_s_* on the maximum values of the gains *R_i_* and *K_v_*. Figure 10 demonstrates the mutual relationship between the two gain values. In accordance with Figure 10b, throughout this paper safe gain values of *R_i_* = 15 Ω and *K_v_* = 0.3 1/Ω are used.

## 7. MATLAB’s Simulink Simulation of MISO PBC Control

**Figure 11:**
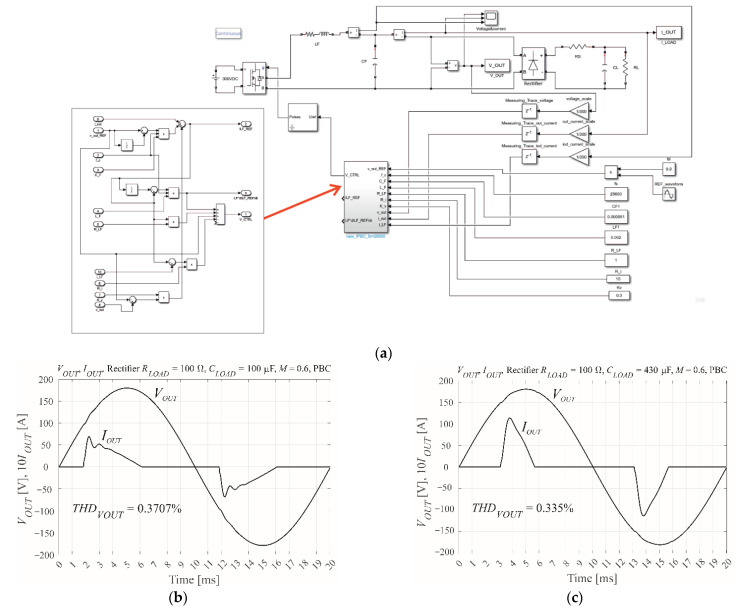
Overview of PBC control, showing (**a**) the MATLAB’s Simulink simulation model of the IPBC2 controller and the simulation model of the inverter with the nonlinear rectifier RC load, (**b**) the simulated output voltage and current with *R* = 100 Ω, *C* = 100 μF, and *M* = 0.6, and (**c**) the simulated output voltage and current with *R* = 100 Ω, *C* = 430 μF, and *M* = 0.6.

Figure 11 presents the Simulink simulation model. The THD is very low, with the MISO-PBC controller (33), and (34) perfectly damping disturbances in the output voltage. The modulation coefficient is less than unity to allow the control voltage to increase. The results of the simulation are ideal; the quantity of THD present is almost negligible. This is a result of using the currents as controller inputs. The scaling coefficient for each of the output voltage, output current, and inductor current is simply 1/*V_DC_*. The modulation index is *M* = 0.6.

## 8. Interfacing MATLAB’s Simulink and dSpace Simulation of the MISO PBC Controller with the Experimental Model

**Figure 12:**
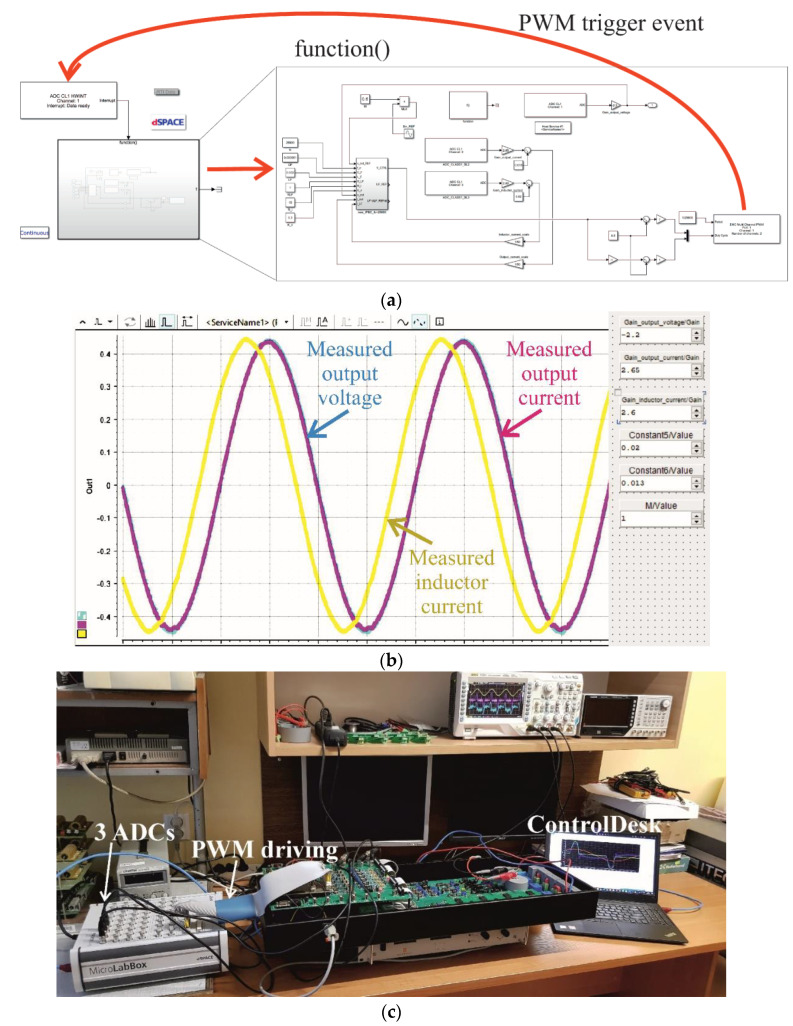
The MicroLabBox real-time interface between the dSpace simulation and the experimental VSI, showing (**a**) the combined Simulink and dSpace simulation model for MicroLabBox (RTI1202) operation, (**b**) scaling of the measuring traces using ControlDesk, and (**c**) an image of the experimental setup showing the three ADCs, the PWM driver, and ControlDesk running on a laptop.

Figure 12a shows the combined Simulink and dSpace models. Shown in Figure 12c, three independent ADCs are used for the output voltage, the output current, and the inductor current. Each ADC is triggered by PWM events. The gain values of *R_i_* = 15 Ω and *K_v_* = 0.3 1/Ω are the same as the Simulink model (Figure 11). The modulation index is *M* = 0.6. However, new scaling of the three measured signals is required. For open loop control with a load of nominal resistance 50 Ω and minimum output capacitance of *C_F_* = 1 μF, which ensures that currents *I_OUT_* and *I_LF_* are approximately equal at the 50 Hz harmonic, the voltage and current traces should have sufficient amplification that they are equal to the reference voltage. The amplification includes the gain of the experimental model measuring traces. The reference voltage is given by 0.5sin(2π50*t*), as shown in Figure 12b. Note that although the figure shows an amplitude of 0.45, the maximum amplitude is 0.5. Finally, the current values are divided by the value of *R_NOM_*: 50 Ω in the presented case. Experimental model measurements [10] show that the delay of the measuring traces and the PWM modulator at *f_s_* = 25,600 Hz can be omitted when designing the controller. ControlDesk software (Figure 12b) was used to tune the scaling coefficients of the measuring traces. The final gain values were −2 for output voltage, 2.65/50 for output current, and 2.60/50 for inductor current.

**Figure 13:**
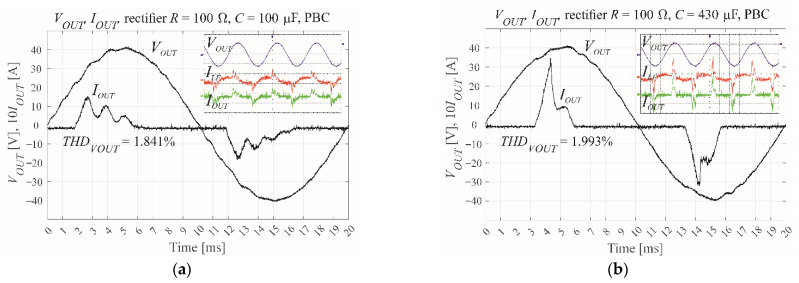
The PBC-controlled VSI output voltage and current as measured in-system using MicroLabBox for a nonlinear rectifier RC load with (**a**) *R* = 100 Ω, *C* = 100 μF, and *M* = 0.6, and (**b**) *R* = 100 Ω, *C* = 430 μF, and *M* = 0.6.

Figure 13 shows the distortions in the output voltage of the VSI when controlled by MicroLabBox. Unlike for SISO-CDM control (Figure 7), the current waveforms are shaped accurately. This is due to MISO control of the output and inductor currents.

## 9. Implementation of the MISO PBC Controller in the VSI Microprocessor 

**Figure 14:**
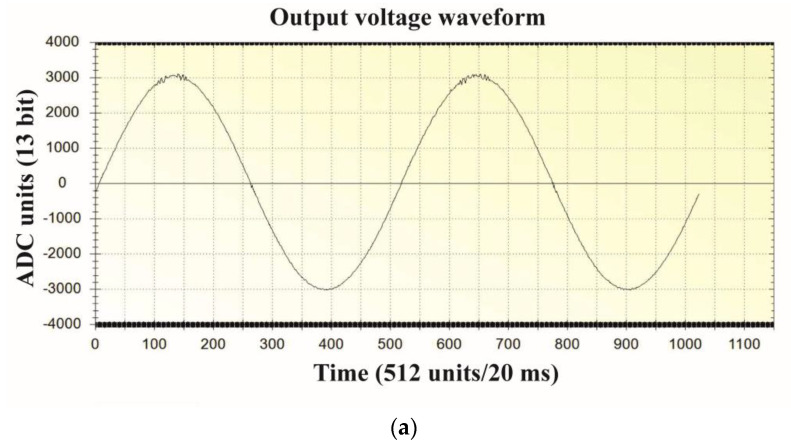

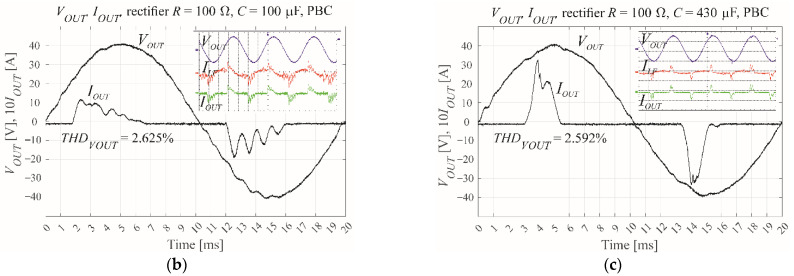
Overview of the VSI as controlled by IPBC2 via the STM32F407VG microprocessor, showing (**a**) scaling of the output voltage measurement, (**b**) measured output voltage and current with *R* = 100 Ω, *C* = 100 μF, and *M* = 0.6, and (**c**) measured output voltage and current with *R* = 100 Ω, *C* = 430 μF, and *M* = 0.6.

The STM32F407VG microprocessor was chosen due to its fast clock speed of 168 MHz, in addition to its 84 MHz maximum PWM comparator input frequency. The microprocessor is capable of floating point hardware operation and has three independent ADCs that can be simultaneously used to measure the output voltage, output current, and inductor current. The amplitude of the reference voltage is 0.5*f_COMPmax_*/*f_s_* = 0.5 × 84 MHz/25600 ≈ 1640. The ADCs obtain measurements in the range −4095–4095. During scaling the system should function under open loop control, with a small output capacitance of *C_F_* = 1 μF and a nominal resistive load of 50 Ω. When using the dedicated PC application to transmit data from the VSI the output voltage is hardware adjusted to 3000 units. Hence, the voltage scaling coefficient is 1640/3000 = 0.547. The current values vary, and as such, they are tuned to a lower value of 2000, from within the −4095–4095 range. With a resistive load of 50 Ω, the current scaling coefficient is (1640/2000)/50 = 0.0164. The modulation index is *M* = 0.6. Figure 14 shows the scaling of voltage measurements, in addition to the measured output voltage and current of the VSI. Control of the current substantially affects the shape of the current waveforms, and reduces distortion of the output voltage.

## 10. Results

The hardware model presented above used the MATLAB’s Simulink 2021b and dSpace Release 2021b along with ControlDesk v.7.5 for MicroLabBox real-time interface 1202, or Keil μVision 5 for STM32F407VG microprocessor control, and a dedicated PC application for USB data exchange that was developed in-house using Microsoft Visual Studio C++ 2019. This hardware model, together with a MicroLabBox RTI1202 real-time interface, were used in the design process of two different VSI controllers: one SISO control system and one MISO control system. The results of using these controllers were evaluated by measuring the THD of the output voltage of the simulated or experimental VSI, when subject to a standard (EN62040) nonlinear rectifier RC load with *R* = 100 Ω, *C* = 100 μF, or *C* = 430 μF, and *PF* = 0.7. The modulation index was reduced to *M* = 0.6 to prevent the modulator from being saturated by rapid increases in the load current. The control procedures of both the real-time interface and the microprocessor were called by PWM block interrupts. For both control systems, the fully simulated system provided better results (lower THD) than the systems that utilized the experimental VSI. One reason for this could be an inaccurate discrete model of the inverter plant within the control design. The CDM control was based upon this model, with linearized functions of the output voltage, output current, inductor current, and duty ratio. Another possible reason could be the approximation of the measuring traces using only the delay values. 

Despite lower performance than the simulated system, the results of the experimental system were satisfactory, and exceeded the requirements of EN 62040-3. Measuring both the output and inductor current allows the MISO controller to accurately shape the output current waveform. This study demonstrates the importance of the precise scaling of voltages and currents. The scaling values differed across each stage of the design procedure and required different procedures at each stage to tune them accurately. This included the use of dedicated software when working with the experimental model controlled by a microprocessor. The relatively low modulation index is important to avoid saturation and enable a faster increase of the inductor current in case of a rapid increase in the load current increase. To this end, the product of the modulation index and the filter inductor inductance should be limited. Table 1 summarizes the findings for each system. The results of the MicroLabBox and microprocessor control procedures are very similar, with a difference in THD of less than 1%.

## 11. Conclusions

This paper detailed the four stages of VSI control system design (Figure 1): development of the theoretical background, modelling, and simulation of the system using MATLAB’s Simulink, control of the experimental VSI using dSpace via a MicroLabBox real-time interface, and implementation of the control system on a STM32F407VG microprocessor for direct control of the experimental VSI. Two control systems were used to demonstrate this process: SISO CDM and MISO PBC. The motivation behind the described design process is the assumption that the differential control laws are consistent throughout each stage of development. Control of the MicroLabBox and the microprocessor is based on PWM block interrupts, with the control procedures called once during each switching period. This approach is feasible as the differential control law obtained from the theory is the same for both simulation and hardware implementation. The primary differences between each stage of the design process are the values of the voltage and current scaling coefficients. These values should be precisely tuned for each stage as the coefficients of the control law would be changed by wrongly scaling. ControlDesk was used to tune the MicroLabBox scaling. Scaling of the microprocessor-based system required dedicated software that enabled data transfer from the hardware. This data transfer to PCB was achieved via USB, with the dedicated software working as a digital oscilloscope scaled in the microprocessor ADC units (Figure 14a). The value of the modulation index is very important during each stage of the design process; an excessively large value can cause saturation of the modulator in the case of rapid increases in load current. Lower values of the product of the modulation index and the filter inductor inductance provide faster changes in the inductor current.

## Figures and Tables

**Table 1:** Summary of the degree of THD for the Simulink model, real-time MicroLabBox control, and STM32F407VG microprocessor control.

Control Type; Nonlinear Rectifier Load RC Parameters Modulation Index M	Simulation	MicroLabBox and Experimental Model	Microprocessor and Experimental Model
Open loop, *R* = 100 Ω, *C* = 100 μF	4.51%	4.35%	-
Open loop, *R* = 100 Ω, *C* = 430 μF	6.75%	6.96%	-
CDM, *R* = 100 Ω, C = 100 μF, *M* = 0.6	0.98%	2.01%	2.65%
CDM, *R* = 100 Ω, C = 430 μF, *M* = 0.6	1.57%	3.44%	3.21%
PBC, *R* = 100 Ω, *C* = 100 μF, *M* = 0.6	0.37%	1.84%	2.63%
PBC, *R* = 100 Ω, *C* = 430 μF, *M* = 0.6	0.34%	1.99%	2.59%

## Data Availability

Not applicable.
